# Higher Education Moderates the Effect of T2 Lesion Load and Third Ventricle Width on Cognition in Multiple Sclerosis

**DOI:** 10.1371/journal.pone.0087567

**Published:** 2014-01-27

**Authors:** Daniela Pinter, James Sumowski, John DeLuca, Franz Fazekas, Alexander Pichler, Michael Khalil, Christian Langkammer, Siegrid Fuchs, Christian Enzinger

**Affiliations:** 1 Department of Neurology, Medical University of Graz, Graz, Austria; 2 Neuropsychology & Neuroscience Laboratory, Kessler Foundation Research Center in West Orange, New Jersey, United States of America; 3 Department of Physical Medicine and Rehabilitation & Neurology and Neurosciences, Kessler Foundation Research Center in West Orange, New Jersey, United States of America; 4 Division of Neuroradiology, Medical University of Graz, Graz, Austria; Klinikum rechts der Isar der Technischen Universitaet Muenchen, Germany

## Abstract

**Background:**

Previous work suggested greater intellectual enrichment might moderate the negative impact of brain atrophy on cognition. This awaits confirmation in independent cohorts including investigation of the role of T2-lesion load (T2-LL), which is another important determinant of cognition in MS. We here thus aimed to test this cognitive reserve hypothesis by investigating whether educational attainment (EA) moderates the negative effects of both brain atrophy and T2-LL on cognitive function in a large sample of MS patients.

**Methods:**

137 patients participated in the study. Cognition was assessed by the “Brief Repeatable Battery of Neuropsychological Tests.” T2-LL, normalized brain volume (global volume loss) and third ventricle width (regional volume loss) served as MRI markers.

**Results:**

Both T2-LL and atrophy predicted worse cognition, with a stronger effect of T2-LL. Higher EA (as assessed by years of education) also predicted better cognition. Interactions showed that the negative effects of T2-LL and regional brain atrophy were moderated by EA.

**Conclusions:**

In a cohort with different stages of MS, higher EA attenuated the negative effects of white matter lesion burden and third ventricle width (suggestive of thalamic atrophy) on cognitive performance. Actively enhancing cognitive reserve might thus be a means to reduce or prevent cognitive problems in MS in parallel to disease modifying drugs.

## Introduction

The cognitive reserve hypothesis suggests that greater intellectual enrichment (e.g. educational attainment (EA); occupational attainment, vocabulary knowledge, premorbid cognitive leisure activity) might moderate the negative impact of pathologic brain changes, such as atrophy, on cognition [1]–[7].

In multiple sclerosis (MS), the relationship between MRI-markers of disease-related tissue changes and cognitive status is variable. Although MRI-markers (e.g. brain atrophy, T2-lesion load; T2-lesion load; T2-LL) can partially explain worse cognitive status, such indices only account for about 10-25% of variance in cognition [2], [8].

Previous work suggested greater intellectual enrichment might moderate the negative impact of pathologic brain changes on cognition in MS patients [2]. However, this finding needs replication in other independent cohorts and has been mostly restricted to mitigating effects on global and regional atrophy measures so far. As T2-LL represents another major predictor of cognitive function in MS, we aimed to test the cognitive reserve hypothesis in a large sample of MS patients from a single center (Graz), using atrophy *and* T2-LL data.

## Methods

### 2.1 Ethics statement

The study was approved by the ethics committee of the Medical University of Graz. All participants gave written informed consent.

### 2.2 Patients

Study participants were enrolled from our MS outpatient department. 137 patients participated in the current study (see Table 1 for characteristics). Patients with a diagnosis of a clinically isolated syndrome (CIS) suggestive of MS, or a diagnosis of relapsing-remitting or secondary progressive MS were included. All patients underwent clinical and neuropsychological testing, and a comprehensive MRI examination. Subjects had no current relapse, had not received corticosteroids 6 weeks prior to inclusion, and had no history of serious psychiatric illness (e.g. depression) or other neurologic disorders.

**Table 1:** Descriptive statistics and scores of neuropsychological and clinical testing for MS patients; means and standard deviations (in brackets).

	All	CIS	RRMS	SPMS
N	137	33	92	12
Sex	89 f/48 m	24 f/9 m	59 f/33 m	6 f/6 m
Age (years)	36.4 (10.1)	34.6 (10.1)	35.9 (10.0)	44.4 (7.3)
Education (years)	13.1 (2.6)	13.1 (2.4)	13.2 (2.8)	12.8 (1.9)
Disease duration (years)	7.5 (7.8)	1.2 (2.9)	8.8 (7.6)	14.6 (7.1)
EDSS	2.0 (1.7)	1.2 (1.0)	1.9 (1.4)	5.7 (1.1)
**Cognition**				
SRT	55.6 (10.5)	60.1 (8.4)	54.3 (10.6)	52.4 (11.8)
SPAT	22.3 (4.9)	23.9 (3.6)	22.1 (5.2)	19.4 (4.7)
PASAT	46.4 (11.1)	48.4 (10.4)	45.4 (11.3)	47.9 (11.4)
SDMT	47.5 (12.7)	52.1 (11.0)	46.7 (13.2)	41.3 (9.2)
WLG	25.9 (6.5)	28.0 (7.4)	25.3 (6.1)	25.4 (6.6)

Legend: N  =  sample size; EDSS  =  expanded disability status scale; SRT  =  selective reminding test; SPAT  =  spatial recall test; PASAT  =  paced auditory serial addition test; SDMT  =  symbol digit modalities test; WLG  =  word list generation; CIS  =  clinically isolated syndrome; RRMS  =  relapsing remitting MS; SPMS  =  secondary progressive MS; f  =  female; m  =  male.

### 2.3 Clinical and Neuropsychological Assessment

Disability was measured using the Expanded Disability Status Scale (EDSS) [9]. Educational attainment (EA) was defined by years of education. Cognition was assessed by the Brief Repeatable Battery of Neuropsychological Tests (BRB-N) [10], comprising the following subtests: 1) Selective Reminding Test (SRT) to assess verbal learning and memory, 2) 10/36-Spatial Recall Test (SPAT) to measure visuospatial learning; 3) the Symbol Digit Modalities Test (SDMT) to measure information processing speed, sustained attention, and concentration; 4) the Paced Auditory Serial Addition Test (3-second version; PASAT) to examine sustained attention and concentration; and 5) the Word List Generation (WLG) to assess semantic verbal fluency (WORD). Results of each subtest were converted to Z-scores to generate an outcome variable for overall cognitive function (composite Z-score; see below).

### 2.4 MRI of the Brain

All MRI examinations were performed at the same 3 Tesla TimTrio scanner (Siemens Healthcare, Erlangen, Germany) using a 12-element receiver head coil and GRAPPA as parallel imaging technique with an acceleration factor of 2. High-resolution structural 3D images were acquired by a T1-weighted MPRAGE sequence with 1 mm isotropic resolution (TR = 1900 ms, TE = 2.19 ms, 176 slices) to assess global atrophy (normalized brain volume; NBV) and regional atrophy (third ventricle width; TVW; mostly indicative of thalamic volume changes) [11]. A fluid-attenuated inversion recovery (FLAIR) sequence with 1×1×3 mm^3^ resolution served for the assessment of T2-LL (TR = 9000 ms; TE = 69 ms, 44 slices).

### 2.5 Image Analyses

All image analyses were done by trained and experienced analysts, blinded to clinical information. T2-lesion load was assessed by a semi-automated region growing algorithm (DispImage) [12]. High-resolution T1 scans served to determine NBV using SIENAX (Structural Image Evaluation, using Normalization, of Atrophy; Version v 2.6, part of fMRIB's Software Library). TVW was measured by the distance in millimeters between the left and right boundaries of the third ventricle on axial T1 images, as previously described [13] (see Table 2 for MRI metrics).

**Table 2:** MRI-metrics for all patients and for each patient group; means and standard deviations (in brackets).

MR-metrics	All	CIS	RRMS	SPMS
T2-LL	16.9 (18.5)	8.0 (9.1)	19.6 (20.5)	21.5 (13.2)
NBV (cm^3^)	1597.5 (99.4)	1647.4 (78.1)	1589.1 (100.7)	1518.0 (75.6)
TVW	4.3 (2.2)	3.4 (1.6)	4.6 (2.3)	4.6 (2.5)

Legend: T2-LL  =  T2-lesion load; NBV  =  normalized brain volume; TVW  =  third ventricle width; CIS  =  clinically isolated syndrome; RRMS  =  relapsing remitting MS; SPMS  =  secondary progressive MS.

### 2.6 Statistical Analysis

Clinical and neuropsychological data were analyzed with the Statistical Package of Social Science (IBM SPSS Statistics 20, SPSS Inc., Chicago, Illinois, USA). The level of significance was set at 0.05. Preliminary analysis (Pearson correlation, point-biserial correlation) examined the relationship between demographic and clinical variables (e.g. sex, age, disease duration) on cognition (dependent variable). We controlled for any demographic and clinical variable significantly correlating with cognition. We checked for fulfillment of different assumptions for the regression analyses (e.g. linearity, homoscedasticity, auto-correlation (Durban-Watson-test), multicollinearity (Variance Inflation Factor)). Hierarchical regression models then served to identify the strongest MRI-predictor for cognitive function. To achieve this, we included sex, age, and disease duration in the first step and individual MRI metrics in a second step.

To assess whether EA moderates the effect of disease severity on cognition, we controlled for sex, age, disease duration in step 1, and in a step-wise manner included MRI metrics (step 2), EA (step 3) and the interaction between EA and MRI-parameters (step 4) in the model (see Table 3). We centered predictor variables to lessen the correlation between the interaction (EA x MRI-parameter) and its component variables. Standardized beta-values (*βj*), adjusted R^2^ (explanation of variance) and delta (Δ) adjusted R^2^ (displaying incremental explanation of variance) in percent are presented for each model in the results section.

**Table 3:** Prediction of Cognition.

MR-metrics	R^2^ (Δ R^2^) MR	R^2^ (Δ R^2^) EA	R^2^ (Δ R^2^) interaction
T2-LL	26.9 (15.2)	33.0 (6.1)	35.2 (2.2)
TVW	14.3 (2.6)	19.4 (5.1)	22.8 (3.4)
NBV (cm^3^)	18.4 (6.7)	23.1 (4.7)	ns

Legend: Adjusted R^2^ and Δ adjusted R^2^ (in brackets) in percent (controlled for age, sex, disease duration) of individual models including MRI-parameters (MR), educational attainment (EA) and interaction (EA x MRI-parameters) (N = 130). T2-LL  =  T2-lesion load; NBV  =  normalized brain volume; TVW  =  third ventricle width; ns  =  not significant.

In subsequent analyses we used “cognitive efficiency” (composite Z-score of SDMT and PASAT) and “memory” (composite Z-Score of SRT and SPAT) as dependent variables to also assess whether EA moderates the effect of disease severity on more specific domains of cognition.

## Results

Table 1 gives information on the descriptive variables and the cognitive and clinical profile of the study cohort, table 2 presents the results for MRI metrics. As can be seen all data are quite in line with what would be expected for the different stages of the disease.

### 3.1 Univariate correlations for cognition

Cognition (defined as the composite Z-score of all subtests of the BRB-N for this and the subsequent analyses unless otherwise stated) significantly correlated with age (*r* = −0.32; *p*<0.001) and disease duration (*r* = −0.30; *p*<0.001). Furthermore, cognitive function differed between men and women (*t*_133_ = −2.17; *r* = 0.19; *p*<0.05).

### 3.2 MRI-predictors of cognition in regression models

Results of the prediction models of cognitive function are presented in table 3.

A regression model including clinical, demographic variables, and T2-LL explained 26.9% of the variance in cognition (T2-LL: Δ R^2^ = 15.2%, p<0.001).

A regression model including clinical, demographic variables, and NBV (global atrophy) explained 18.4% of variance (NBV: Δ R^2^ = 6.7%, p<0.05).

A regression model including clinical, demographic variables, and TVW (regional atrophy) explained 14.3% of variance of cognition (TVW: Δ R^2^ = 2.6%, p<0.05).

### 3.3 Regression models testing the cognitive reserve hypothesis

The full T2-LL regression model, including clinical and demographic variables, as well as T2-LL, EA and their interaction (EA x T2-LL), explained 35.2% of the variance of cognition.

There was a positive effect of sex (*βj* = 0.21, *p*<0.05; male  = 1; female  = 2; women performed better than men) in step 1 (R^2^ = 11.7%), a negative effect of T2-LL (*βj* = −0.48, *p*<0.001) in step 2, and a positive effect of EA (*βj* = 0.26, *p*<0.001) in step 3.

**Figure 1:**
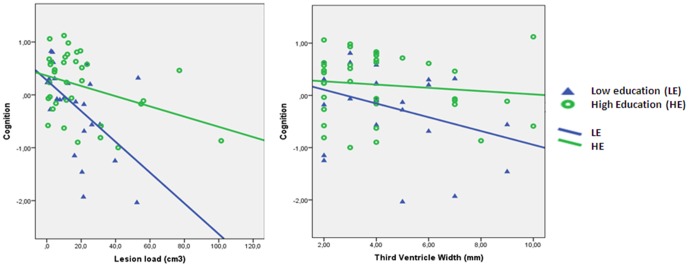
Correlation of lesion load/third ventricle width with cognition for high and low education. Correlation for low education (25^th^ percentile: ≤11 years of education; blue triangles) *r* = −0.52; *p*<0.05/*r* = 0.33; *p*<0.05 (N = 27) vs. correlation for high education (75^th^ percentile: ≥14 years of education) *r* = −0.37; *p*<0.05/*r* = 0.12; *ns* (N = 36), including fit of linear regression lines.

Furthermore, the interaction between EA and T2-LL on cognition was significant (*βj* = 0.17, *p*<0.05; see Table 3), whereby higher EA (as assessed by years of education) moderated/reduced the negative effect of T2-LL on cognition (Figure 1).

The full NBV regression model explained 23.1% of cognition. NBV (*βj* = 0.30, *p*<0.001) and education (*βj* = 0.23, *p*<0.05) had a positive effect on cognition. Here, no significant influence of the interaction term was observed (*βj* = −0.07, *p* = 0.37).

The full TVW regression model explained 22.8% of the variance of cognition (see Table 3; Figure 1). There was a positive effect of sex (*βj* = 0.24, *p*<0.05; women performed better than men) in step 1, a negative effect of TVW in step 2 (*βj* = −0.24, *p*<0.05), and a positive effect of EA (*βj* = 0.22, *p*<0.05) and the interaction in step 3 and 4, respectively. Higher EA moderated/reduced the negative effect of TVW on cognition (*βj* = 0.21, *p*<0.05).

Subsequent analyses revealed that 31.1% of variance of cognitive efficiency was predicted by a regression model including demographic and clinical variables, T2-LL, EA and the interaction. After controlling for clinical and demographic variables (R^2^ = 6.3%), T2-LL still negatively affected cognitive efficiency (Δ R^2^ = 17.5%), EA was positively correlated with cognitive efficiency (Δ R^2^ = 5.5%) and the interaction between EA and T2-LL explained incremental 1.8% (*βj* = 0.16, *p*<0.05). No significant interactions were observed if memory was used as dependent variable.

No significant interaction for specific cognitive domains (cognitive efficiency and memory) was observed including NBV or TVW in the models.

## Discussion

Higher educational attainment (as assessed by years of education) attenuated the negative effect of MS-related disease burden, measured by T2 lesion load and regional atrophy (TVW), on cognition. This corroborates and extends previous findings, reporting that cognitive reserve moderates the negative impact of pathologic brain changes on cognition in the context of MS [2], [4], [13].

Although lesion load is known to be an important predictor of cognitive function in MS [14], most studies investigating the cognitive reserve hypothesis so far only used global or regional brain atrophy to assess disease burden in MS patients. Hence, we included both lesion load and atrophy to predict cognitive function and assess the incremental predictive value of EA, as well as the protective effect of cognitive reserve in MS patients (i.e., interaction effect). In our sample, lesion load proved to be the strongest predictor of cognitive function and higher EA protected against cognitive decline related to higher lesion load.

Furthermore, higher EA lessened the negative impact of regional atrophy (TVW, suggestive of thalamic volume loss) on cognitive function. Prior studies highlighted subcortical grey matter atrophy, especially thalamic atrophy [15], to be strongly associated with cognitive function in MS, suggesting central rather than global brain atrophy to be strongly associated with cognitive impairment in MS [8].

Although global atrophy was associated with cognitive function in our study, in contrast to previous studies [5], cognitive reserve did not attenuate the negative effects of global atrophy on cognitive function in our sample. One possible explanation may be that regional atrophy precedes global atrophy as a marker for cognitive dysfunction. Consistent with this notion, the mean disease duration in our sample was relatively short, i.e. 7.5 years, compared to e.g. Amato et al. [5] who reported modified associations between global atrophy and cognition due to cognitive reserve in an MS sample characterized by an average disease duration of 11 years. Also several of our patients were still in the stage of CIS. It is conceivable that, the destruction of more critical and specific brain regions is critical for in cognitive alterations [16] before a more general decrease in brain volume occurs. The mitigating effect of EA against global atrophy may therefore become more evident at later stages of the disease only.

Importantly, cognitive reserve represents a potentially modifiable variable that could reduce or prevent cognitive problems in MS [17], although most recent investigations suggest that the effect of this might diminish with progression of disease.^5^ We found that in addition to MRI-metrics, educational attainment incrementally explained around 5–6% of variance of cognition.

Similar to Sumowski et al. [2] who found that the interaction of intellectual enrichment and brain atrophy explains 5 to 7% of variation after accounting for the direct influences, cognitive reserve accounted for 3–4% of variance in our sample. It is of note that the effect of cognitive reserve appears to be strongly dependent on the educational level of the study sample. Indeed, the educational level of the sample in Sumowski et al. 's study was rather high (mean EA = 16 years), compared to ours (mean EA = 13 years), which would fit with a somewhat higher mitigating effect in the Sumowski et al. study. Furthermore, for linear regression models large sample size (N>100) are recommended to avoid overestimation of R^2^. Hence, although support for the cognitive reserve hypothesis is steadily increasing [4], [5], [13], [18], the significance and size of the effect of cognitive reserve still remains to be more thoroughly investigated.

We observed a positive effect of sex (women performed better than men) for overall cognition. There are three possible explanations for this finding. First, general gender related differences in cognitive function irrespective of the disease might play a role [19]. Secondly, possible gender differences in the cognitive sequeale of MS might exist [20]. Third, the higher prevalence of women with MS [21] might further influence the finding through increased variance.

Although the main focus of our study was laid on general cognitive functioning, we subsequently assessed whether higher EA also moderated the effect of more specific cognitive domains (i.e., cognitive efficiency or memory). We found that higher educational level (as assessed by years of education) specifically protected from the negative impact of higher T2-LL on the domain cognitive efficiency. Interestingly, no protective effect of EA was observed for regional atrophy (TVW). Although the thalamus seems to be essential for general cognitive function, cognitive efficiency thus might be related to different brain pathologies, e.g. the contribution of frontal white matter lesions. Further studies in different samples are clearly needed to more specifically address and clarify this question. No mitigating effect of higher EA was observed for the memory domain.

Furthermore, we found no significant interaction for specific domains (cognitive efficiency and memory) including NBV or TVW in the models. This might be due to a decreased variance in individual domains compared to overall cognitive function.

Some limitations of our study have also to be considered. Intellectual enrichment was only assessed here by EA. The use of additional indices of intellectual enrichment (such as occupational attainment, premorbid intelligence, leisure activities, etc.) would have provided a more complete estimation of cognitive reserve. However, EA provides a robust and objective estimate of intellectual enrichment [22] and there exist strong correlations between the different indices of intellectual enrichment. Furthermore, we did not assess regional lesion volumes which might be important for more detailed analyses of the mitigating effects of EA on specific cognitive domains. However, this would necessitate an even larger and more diverse patient cohort. Further studies should be planned to investigate how the localization and distribution of lesions might account for specific cognitive impairments, and if these impairments could be attenuated by cognitive reserve. Furthermore, our results cannot be safely extrapolated to the whole spectrum of MS patients, as our study sample mainly consists of ambulatory patients eligible for disease modifying treatment seen at our MS-outpatient department (24% CIS, 67% RRMS, 9% SPMS). Also, several other parameters such as life-style and risk factors (such as smoking or alcohol consumption) might have influenced our findings, as evidenced by e.g. previous work in healthy individuals suggesting that life-style affects atrophy [23].

Practical implications of our findings are twofold. First, EA needs to be considered in any investigation of the association between morphologic brain changes and cognition in MS. Second, cognitive reserve obviously represents a potentially modifiable variable that could reduce or prevent cognitive problems in MS [17]. Thus, early identification of MS patients at greater risk for cognitive decline (i.e., those with lower levels of EA) could be valuable to delay the progression of cognitive problems through therapeutic interventions [24] (e.g. cognitive training). Clearly, treatment with disease modifying drugs (DMD) is still the most crucial factor to reduce disease progression, but actively enhancing cognitive reserve might be a means to reduce or prevent cognitive problems in MS in parallel to treatment with DMD. Future studies thus should embark on investigating the potentially beneficial effects of actively increasing intellectual enrichment in our patients with MS.
